# Muons, mutations, and planetary shielding

**DOI:** 10.3389/fspas.2022.1067491

**Published:** 2022-11-23

**Authors:** Piet C. de Groen

**Affiliations:** University of Minnesota, Minneapolis, MN, United States

**Keywords:** muons, magnetospheres, exoplanets, DNA, mutations, evolution, aging, diseases

## Abstract

Life on earth is protected from astrophysical cosmic rays by the heliospheric magnetic and slowly varying geomagnetic fields, and by collisions with oxygen and nitrogen molecules in the atmosphere. The collisions generate showers of particles of lesser energy; only muons, a charged particle with a mass between that of an electron and a proton, can reach earth’s surface in substantial quantities. Muons are easily detected, used to image interior spaces of pyramids, and known to limit the stability of qubits in quantum computing; yet, despite their charge, average energy of 4 GeV and ionizing properties, muons are not considered to affect chemical reactions or biology. In this Perspective the potential damaging effects of muons on DNA, and hence the repercussions for evolution and disease, are examined. It is argued here that the effect of muons on life through DNA mutations should be considered when investigating the protection provided by the magnetic environment and atmosphere from cosmic rays on earth and exoplanets.

## 1 Introduction

### 1.1 Planetary Shielding

The magnetic structure of the heliosphere and the magnetic field of the Earth’s magnetosphere are important for controlling the fluxes of cosmic rays that reach the upper atmosphere, and the variability of those fields means that cosmic-ray fluxes differed in the past and will differ in the future. (Potgieter, 2013). Cosmic rays lead to mutations in DNA, which may enable evolution but also can be life threatening. The effects of cosmic rays are not limited to earth; indeed, potential shielding provided by magnetic environments of exoplanets in habitability zones around other stars is a constant topic of study (Grießmeier et al., 2005; Metcalfe et al., 2013; Farrish et al., 2019; Mesquita et al., 2021).

### 1.2 Muons

Due to special relativity and collisions with oxygen and nitrogen molecules in the atmosphere, the main cosmic rays-derived air showers at sea level consist of muons, charged particles with a mass between that of an electron and a proton. (Einstein, 1905; Allkofer et al., 1971; Allkofer et al., 1985; Rastegarzadeh and Khoshabadi, 2016). Although muons can be detected with simple instruments, allow imaging of interior spaces of pyramids and shipping containers, and interfere with stability of qubits in quantum computing, muons are dismissed as a cause of or affecting chemical reactions in biology. (Vepsalainen et al., 2020; Adam, 2021; DOE Explains…Muons, 2022). In this Perspective/Opinion paper, the point will be made that cosmic ray-derived air showers are critical for life as it exists on earth. Evolution and support of water/carbon-based life on exoplanets therefore will require not only a similar extended magnetosphere, an orbit with low eccentricity to prevent extreme temperatures, but also an atmosphere protecting its surface from high-energy cosmic ray particles yet allowing enough muons to reach its surface for life to evolve.

## 2 How mutations work

Evolution of life requires constant mutations in DNA, the molecule that carries genetic information for the development and functioning of organisms. The process of DNA mutation is poorly understood.

A mutation is an alteration in the nucleotide sequence of a DNA molecule. The mainstream theory poses that mutations result either from errors in DNA replication or from the damaging effects of mutagens, such as chemicals and radiation, which react with DNA and change the structure of one or more of the four different types of DNA nucleotides (Brown, 2002). There is no explanation for an occasional error in the chemical reactions involved in DNA duplication; the exchange of electrons, formation of new bonds, and energy requirements are identical for each successive reaction. Yet, the molecular explanation for DNA mutations is “random error” or when it occurs in repetitive DNA sequences “slippage”. Random error is hypothesized to occur when the DNA polymerase temporarily dissociates from the DNA template during DNA duplication and slippage is hypothesized to occur due to re-annealing after dissociation to a similar repetitive sequence at a different position one or more repeats up- or down-stream from the site of dissociation (Ellegren, 2004). Random and slippage errors are postulated to account for 50–75% of the risk for many diseases.

There are two main types of DNA damage, one involving a single strand of the DNA double helix (single-strand break) and the other affecting both strands (double-strand break) (Chang et al., 2017). Single-strand DNA breaks are common and easily repaired using the non-damaged strand as template. Double-strand DNA breaks can have unpaired, overhanging ends, where the two strands are damaged at different locations within the double helix; when few nucleotides are missing, and the overhanging ends consists of non-repetitive nucleotide sequence error-free repair is likely. Finally, double-strand breaks that affect the DNA helix strands at the same location do not have overhanging ends and are known as blunt-end DNA; if nucleotides are missing, a simple reconnection of the blunt ends results in a mutation. The only way to repair this type of damage without errors is *via* a complex, cell cycle-dependent mechanism involving the complementary chromosome as template. Therefore, double-strand, blunt DNA breaks are most likely to result in mutations.

The mainstream theory is problematic. DNA replication occurs in proliferating cells thus there have been efforts to tie DNA mutation directly to the proliferation rate of tissues (Tomasetti et al., 2017). But many cells prone to mutations do not have a high proliferation rate, or—as in neurons—no longer proliferate at all. To add to the problem, many of these cells are not exposed to known chemicals or radiation. Moreover, for some hereditary diseases it is known that inherited mutations in specific DNA loci increase the risk for disease; somatic, i.e., acquired during life, mutations in the same specific loci in persons without inherited mutations cause sporadic disease identical to the inherited disease (Miyaki et al., 1994; Aitchison et al., 2020). Thus, mutations are not truly random but instead associated with mutation-prone DNA such as “hotspots”: DNA sequences or loci in the genome in which mutations arise at a higher rate than would be predicted if mutations were truly random (de Groen, 2022a; de Groen, 2022b). Yet, despite all these issues with the mainstream theory no new explanations as the cause of DNA mutations are being considered.

## 3 Muons and DNA damage

**FIGURE 1:**
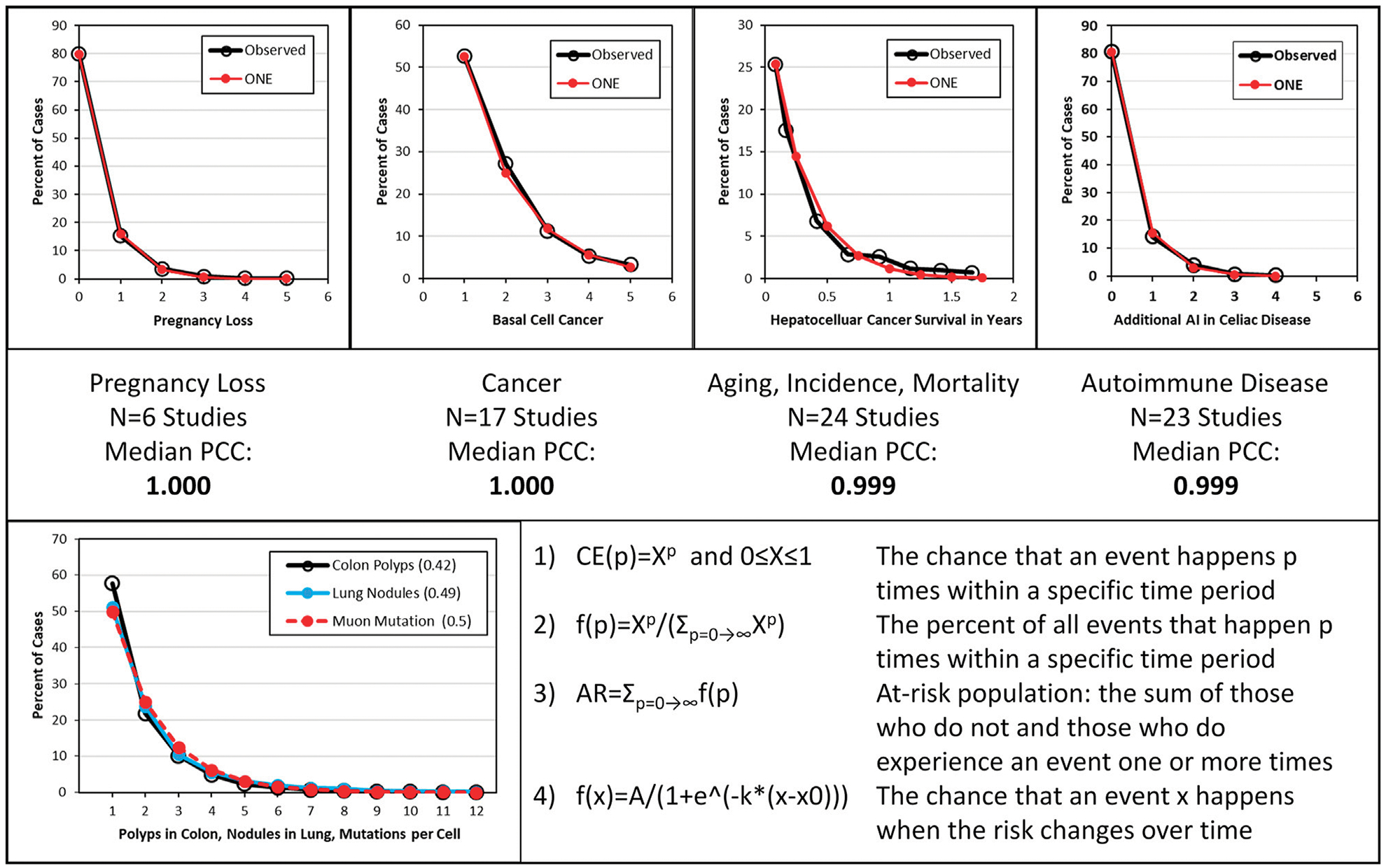
Examples representing 70 studies, the associated Pearson correlation coefficients, a comparison of polyp, lung nodule and estimated muon event distributions and the omnipresent neoplasia equations. (Heuvelmans et al., 2017; Xirasagar et al., 2020; de Groen, 2022c). Top: Examples of distribution of one or more disease events per person. From left to right are shown spontaneous pregnancy loss, cancer, aging, disease incidence and mortality, and autoimmune disease. “Observed” depicts documented data; “ONE” depicts modeled data using omnipresent neoplasia equation 2) derived from colon polyp studies and shown in the bottom panel. Middle: A summary of the type of disease, the number of studies and the median Pearson correlation coefficient (PCC) shown in Top panel. Bottom: Left: Observed colon polyp and lung nodule data in at-risk persons around age 60 and mutations per cell in 0.5 days given 1 muon event/cell/day. Right: omnipresent neoplasia equations (de Groen, 2020a).

The number of polyps detected per endoscopist in patients undergoing colonoscopy to prevent colorectal cancer was found to adhere to an exponential pattern; the accepted, underlying cause for polyps is DNA mutations (de Groen et al., 2020). The same pattern was present in many other diseases manifested by multiple events (Figure 1); this supports known or suspected DNA mutations as underlying cause of these diseases (de Groen, 2022c). Because the same pattern can be detected in neurodegenerative diseases due to DNA mutations in terminally differentiated neurons which no longer can replicate, there must be a constant source of DNA damage causing mutations in the absence of DNA replication.

DNA is mostly damaged by chemical and radiation sources. Chemical sources can be discarded given the great variation of exposure to chemicals among persons now and during evolution. Among radiation sources, medical sources have only been present during the last century, and their use varies widely among countries. Radon and Thoron do not have the tissue penetration or energy in eV to explain diseases other than lung cancer. Internal, terrestrial, consumer, industrial and occupational ionizing radiation exposure also does not explain the observations as shown in Figure 1. Thus, high linear energy transfer radiation from medical sources, soil, buildings, food-derived particles, Radon and Thoron do not encompass the required timespan of exposure, equal distribution among all persons, tissue penetration, bodily distribution, or energy scattering characteristics. The only type of radiation remaining is minimum ionizing particle (MIP) radiation, which consist mostly of muons.

A key question is whether muons can and do damage DNA. Muons have four critical characteristics that explain their effect on DNA within living cells at earth’s surface level. First, muons have the energy to cause direct DNA damage. Moreover, they can produce high-energy knock-on electrons and bremsstrahlung resulting in a shower of secondary particles. With each particle-particle collision more particles of lesser energy may form. DNA damage in an aqueous environment occurs mostly through formation of hydroxyl radicals and hydrated electrons which leads to reactive oxygen species, a universally accepted, common cause of chemical additions to DNA, single- and double-strand DNA breaks. A single electron of 30 eV or greater can be involved in multiple successive interactions with supercoiled DNA causing multiple double-strand DNA breaks (Huels et al., 2003). A single muon of 4 GeV passing through 20 cm of aqueous tissue will lose about 40 MeV or 1% of its energy as an extensive shower of particles of lesser energy including many electrons: enough to cause more than one million double-strand DNA breaks (NASA, 2022; Interaction Depth, 2022). If we assume an average body surface area in recumbent position of 0.35 m^2^, a body height in that position of 20 cm, a body density of 1 g/cm^3^, a muon flux of 14.4 million muons per m^2^ per day, a muon energy loss of 2 MeV per g/cm^2^ and a 30 eV requirement per mutation then enough muon energy to cause at least 6.7 trillion mutations scatters throughout a human body every day. Assuming a 5 eV requirement per single double-strand DNA break and 37 trillion cells per human body, then enough energy for 40 trillion mutations or one mutation per cell is absorbed by an adult person on a daily basis (Bianconi et al., 2013). The distribution of muon events among cells can be modeled by the same exponential equation as used to model the distribution of disease events (Figure 1). (Heuvelmans et al., 2017; de Groen, 2020a; Xirasagar et al., 2020; de Groen, 2022c)

Second, the energy spectrum from muons of variable eV and the cascade of decay products, including muons of lower energy, hydroxyl radicals, and electrons of varying eV, cover the required range of energies to cause destruction of the normal chromosome pattern during cell division, point mutations, translocations, insertions, deletions, and compound DNA mutations, and chemical breaks and dissociations in other molecules. From here forward the term “muon event” will be used to indicate any of these forms of muon-related damage. Immature eggs remain in an early stage of meiosis between birth and ovulation; in this timespan they are exquisitely sensitive to radiation (Hanoux et al., 2007). If an extensive shower of muon events damages hundreds of egg cells, the outcome will be a combination of simple DNA mutations or, when damage is more severe, programmed cell death (Hanoux et al., 2007). The expected egg cell depletion will be large early in life (80% depletion before first occurrence of menstruation) and exponentially taper toward menopause—which is exactly what has already been described in 1963 (Baker, 1963; de Groen, 2020b). In regard to cancer, only a single cell needs to undergo a set of specific somatic mutations to start colorectal cancer and as few as three critical mutations appear sufficient to initiate this disease (Barker et al., 2009; Vogelstein and Kinzler, 2015). Using muon properties as listed above, one can calculate that the chance for colorectal cancer of a person with a dominant familial adenoma-type colon polyp syndrome (FAP) at age 30 is about 1–2X the chance that 1 out of 20 persons without an FAP mutation develops a sporadic colorectal cancer at age 60 (with represents a 5% estimated population risk for sporadic colorectal cancer). This relative risk is in line with population-related relative risks and the nearly 100% lifetime risk for persons with FAP at a median age of 39 (Ellis, 2005).

Third, enough muons need to pass through or decay within a human body during an average lifetime to cause inherited and acquired DNA mutations (Martincorena et al., 2015). We lack solid experimental data to verify whether this is the case. By sequencing two parents and their two children, a DNA mutation estimate of 1.1 × 10^−8^ per position per 23 chromosomes or 70 mutations per 23 pairs of chromosomes was found (Roach et al., 2010). Of the genetic mutations identified, all were simple mutations as expected in DNA derived from living persons. If we assume an average generation timespan of 25 years, an average body cell count of 37 trillion cells, 6.7 trillion mutations per day evenly distributed over all nucleotides and cells, then we expect about 1700 potential mutations per cell after 25 years (Bianconi et al., 2013). As DNA damage per muon and other factors may fluctuate, severe genetic damage will not lead to offspring and most DNA damage will be repaired, this estimation is not incompatible with the observed 70 mutations per genome.

Fourth, unwound, single-strand DNA is most susceptible to damage by ionizing radiation; the damage depends on the actual DNA sequence and for certain hotspots is increased in the presence of enzymes which destabilize DNA through cytidine deaminase activity (Weissberger and Okada, 1961; Gonzalez-Fernandez and Milstein, 1993; Wang et al., 2009; Ebel and Bald, 2022). DNA is in unwound, single-strand configuration during cell division and—of critical importance—protein formation. Muon events therefore explain double-strand DNA breaks and mutations in the absence of cell division, for instance in neurons producing proteins in the presence of cytidine deaminase activity.

In conclusion, patterns of DNA mutations observed as patterns of human disease closely adhere to patterns expected under conditions of a constant air shower of muons causing constant DNA damage. Thus, DNA damage by muons explains evolution, aging, and disease (Colchero et al., 2021; Yamamoto et al., 2022).

## 4 Evidence of muons

**FIGURE 2:**
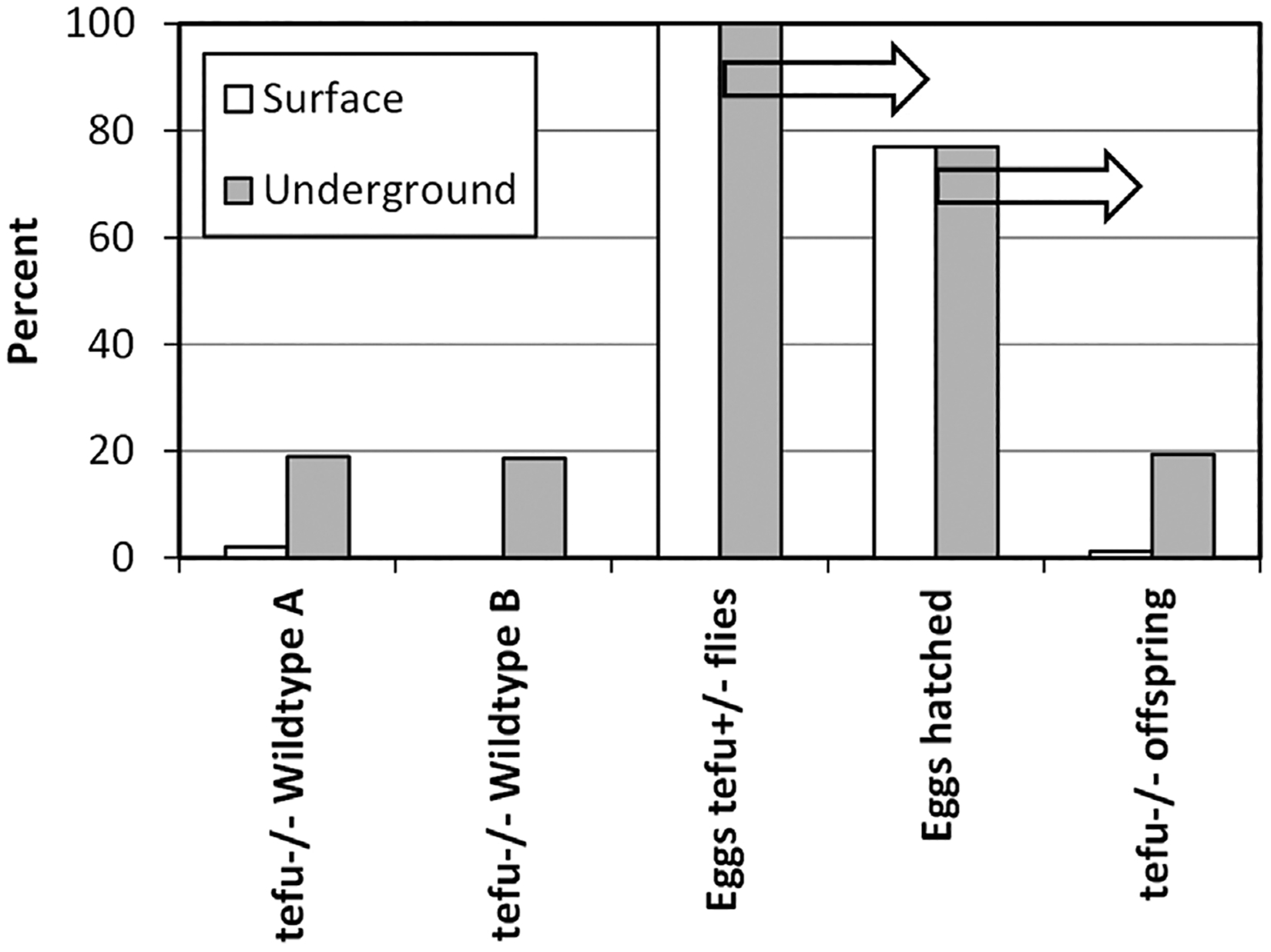
The effect of MIP radiation on survival of fruit flies with *tefu*^−/−^, the fruit fly gene similar to the human ATM gene. At surface level, offspring from heterozygous *tefu*^+/−^ flies consists for about 2% of flies homozygous for *tefu*^−/−^. This effect happens independently of background strain, as shown for wildtype A and B. The mutation does not alter the number of eggs produced or the number of eggs hatched (77% each at surface and underground level), but only deep underground in the absence of nearly all MIP radiation do *tefu*^−/−^ eggs develop into the expected number of fruit flies. Arrows mark sequential experimental results. Adapted from Morciano et al. (2018).

Another key question is whether near absence of muons results in near absence of mutations. Given an energy loss of 2 MeV per g/cm^2^, only a few muons of minimal energy will be remaining deep underground or underwater. A group in Italy studied two groups of fruit flies at surface level and shielded from 99.9% of MIP radiation-related DNA damage, mainly through absence of muons, by 1.4 km of rock in the Grand Sasso underground laboratory (Morciano et al., 2018). One group of flies exhibited normal DNA repair, and one group was heterogeneous for *tefu*, the fruit fly gene similar to the human ATM gene that is critical for repair of DNA damaged by ionizing radiation and maintenance of normal levels of meiotic recombination in oocytes (Barlow et al., 1998). At surface level barely any *tefu*^−/−^ eggs developed into fruit flies but deep underground, protected from DNA damage by muons and thus without a need for continuous DNA repair, nearly the expected proportion of *tefu*^−/−^ eggs developed into fruit flies (Figure 2). (Morciano et al., 2018) Mariana hadal snailfish are shielded from MIP radiation by more than 6 km of water and, when compared to similar fish species living closer to the sea surface, have a very low mutation rate mostly limited to point mutations across the whole genome over the past 20 million years while the species evolved at the bottom of the Indian Ocean (Wang et al., 2019). At a depth of more than 6 km snailfish are not exposed to high energy MIP events, preventing extensive DNA damage and complex mutagenesis.

## 5 Implications for space physics

The search for exoplanets able to support water/carbon-based life as we know it on earth and the requirements to establish long-term human colonies on the moon or planets within our Solar System will need to include the combination of protection from nearly all cosmic rays-derived air showers and inclusion of MIP radiation such as provided by muons at earth surface to support life and drive evolution. For exoplanets this implies star-exoplanet combinations with a similar extended magnetosphere, exoplanet orbits with low eccentricity, a similar average exoplanet temperature, presence of water and carbon, and an exoplanet atmosphere protecting its surface from high-energy cosmic ray particles yet allowing enough muons to reach its surface for life to evolve.

## 6 Discussion

The evidence suggests that it may be time to discard the term “random error” when it comes to DNA mutations and replace it by “muon event”. The constant need of all life for DNA duplication and protein formation implies a constant state of unwound, single-strand DNA predisposing to a constant risk for particle-based DNA damage and thus a constant rate of DNA mutations. Muon events are independent of cellular proliferation and explain mutations in terminally differentiated cells such as neurons. Heritability, lifestyle, environmental factors, and muon radiation together likely determine 100% of the risk for disease. Muon-based mutagenesis represents the missing evolutional and environmental risk factor, is a common mechanism of disease, preferentially affects mutation-prone DNA, explains a constant need for DNA protection and repair, and may provide novel insight in the basic mechanism of aging and many diseases that are currently poorly understood such as type 1 diabetes, Graves’ disease, Celiac disease, FAP, Alzheimer’s disease, Parkinson’s disease, Huntington’s disease, myotonic dystrophy, and Friedreich’s ataxia. The exponential equations used to model distribution of diseases and muon events connect Darwin to Einstein: evolution and biology are a special form of particle physics restricted to the unique environment provided by the milky way Galaxy, Solar System and planet earth (Darwin, 1859; Einstein, 1905). Searches for exoplanets able to support water/carbon-based life centered around DNA therefore will need to consider a balance of cosmic ray shower shielding with selective passage of enough muons able to reach the planet’s surface to allow formation and evolution of DNA-based life.

## Data Availability

The raw data supporting the conclusion of this article are available within published articles.
